# Glycoprotein YKL-40: a novel biomarker of chronic graft-vs-host disease activity and severity?

**DOI:** 10.3325/cmj.2016.57.239

**Published:** 2016-06

**Authors:** Nadira Duraković, Ivan Krečak, Zinaida Perić, Milan Milošević, Lana Desnica, Dražen Pulanić, Iskra Pusic, Vesna Kušec, Radovan Vrhovac, Steven Z. Pavletic, Damir Nemet

**Affiliations:** 1 University of Zagreb, School of Medicine, Zagreb, Croatia; 2 Department of Internal Medicine, Division of Hematology, University Hospital Center, Zagreb, Zagreb, Croatia; 3 General Hospital Šibenik, Šibenik, Croatia; 4 Faculty of Medicine Osijek, J. J. Strossmayer University of Osijek, Osijek, Croatia; 5 Division of Oncology, Section of Bone Marrow Transplant and Leukemia, Department of Medicine, Washington University School of Medicine, St Louis, MO, USA; 6 Clinical Department of Laboratory Diagnosis, University Hospital Center Zagreb, Zagreb, Croatia; 7 Experimental Transplantation and Immunology Branch, Center for Cancer Research, National Cancer Institute, National Institutes of Health, Bethesda, MD, USA

## Abstract

**Aim:**

To investigate whether increased YKL-40 levels positively correlate with graft-vs-host disease (cGVHD) activity and severity and if YKL-40 could serve as a disease biomarker.

**Methods:**

This case-control study was conducted at the University Hospital Centre Zagreb from July 2013 to October 2015. 56 patients treated with hematopoietic stem cell transplantation (HSCT) were included: 35 patients with cGVHD and 21 without cGVHD. There was no difference between groups in age, sex, median time from transplant to study enrollment, intensity of conditioning, type of donor, or source of stem cells. Blood samples were collected at study enrollment and YKL-40 levels were measured with ELISA. Disease activity was estimated using Clinician’s Impression of Activity and Intensity of Immunosuppression scales and disease severity using Global National Institutes of Health (NIH) score.

**Results:**

YKL-40 levels were significantly higher in cGVHD patients than in controls (*P* = 0.003). The difference remained significant when patients with myelofibrosis were excluded from the analysis (*P* = 0.017). YKL-40 level significantly positively correlated with disease severity (*P* < 0.001; correlation coefficient 0.455), and activity estimated using Clinician’s Impression of Activity (*P* = 0.016; correlation coefficient 0.412) but not using Intensity of Immunosuppression (*P* = 0.085; correlation coefficient 0.296).

**Conclusion:**

YKL-40 could be considered a biomarker of cGVHD severity and activity. However, validation in a larger group of patients is warranted, as well as longitudinal testing of YKL-40 levels in patients at risk of developing cGVHD.

Chronic graft-vs-host disease (cGVHD) remains the most important cause of non-relapse morbidity and mortality in long-term survivors after hematopoietic stem cell transplantation (HSCT) (1,2) and by far its most intriguing complication. Although the precise immunologic mechanism leading to cGVHD development still remains to be elucidated, there have been some recent advances in understanding the disease process and identification of potential biomarkers (1-4). cGVHD is a multisystem disorder characterized by immune-dysregulation, resulting in impaired organ function, increased risk of infections, and deteriorated quality of life. Patients present with a variety of symptoms and organs involved including the skin, mouth, eye, gut, liver, lungs, joints, and genitourinary system (5-7). In the recent years the incidence of cGVHD has been increasing (2), likely related to the increased donor (8) and recipient age (9), decreased early post-transplant mortality, use of matched unrelated donors, and peripheral blood stem cell grafts (10,11). Identifying biomarkers that could be used to predict response to treatment, assess disease activity, or distinguish reversible disease activity from irreversible damage would be of great clinical value (12). Unfortunately, even though a number of potential biomarkers have been identified, such as anti-double-strand DNA antibodies, adiponectin, soluble IL-2 receptor α (IL-2Rα), B-cell activating factor (BAFF), CXCL9, and CD13 (12-15), there is still no reliable marker that could be widely used in cGVHD patients.

Chitin, a polymer of N-acetylglucosamine, is present in coatings and cell walls of many organisms including bacteria, fungi, nematodes, insects, and plants (16-21). Chitinases, whose function is to degrade chitin, have been generally considered not to be present in mammals due to the absence of chitin. YKL-40, a member of the mammalian chitinase-like glycoproteins, is a heparin- and chitin-binding lectin without chitinase activity (22). It is expressed in various cell types including neutrophils (23), macrophages (24), bone marrow megakariocytes (25), chondrocytes and synovial cells (26,27), as well as in malignant cells (28). In normal bone marrow, YKL-40 protein is stored in the granules of the myelocytes and metamyelocytes, and released from fully activated cells (29). YKL-40 is also expressed by macrophages *in vitro* during the late stage of differentiation (29), *in vivo* during inflammation (30), and by peritumoral macrophages (31). Furthermore, YKL-40 modulates vascular endothelial cell morphology by promoting the formation of branching tubules, acts as a chemoattractant for endothelial cells, stimulates their migration, and promotes the migration and adhesion of vascular smooth muscle cells, indicating its role in angiogenesis (32). It has been shown to increase the growth rates of fibroblasts synergistically working with insulin-like growth factor-1 (IGF-1) (26). Its production is regulated by various cytokines. Studies in interleukin 6 (IL-6) knockout mice revealed that YKL-40 expression depended on IL-6 (33). Expression of YKL-40 mRNA in human monocyte is strongly stimulated by IFNγ, and inhibited by IL-4 and dexamethasone (34).

The physiological and biological functions of YKL-40 are still unclear. YKL-40 has been implicated in various inflammatory conditions, such as infections (35), autoimmune diseases (36-38), liver diseases (39,40) and malignant diseases (24,25,28,41-45). Mainly due to its role in inflammation (30) and extracellular matrix remodeling (26), it has been investigated as a potential biomarker of several autoimmune conditions (36,38), as well as those that include fibroblast activation (40,43).

In the non-myeloablative allogeneic HSCT setting, higher pretransplant recipient and donor plasma YKL-40 concentrations suggest a role for YKL-40 as a biomarker for relapse and treatment-related toxicity. Recipients with pretransplant YKL-40 concentrations above the age-adjusted 95th percentile (high) had higher relapse-related mortality and lower progression-free and overall survival. Recipients transplanted with donors with high YKL-40 concentrations had an increased probability and risk of grade 2-4 acute graft-vs-host disease (aGVHD) (45,46). However, none of the studies so far has examined whether post-transplant levels of YKL-40 influence the transplant outcomes or GVHD.

Based on the strong involvement of YKL-40 in inflammatory processes and autoimmune disorders, particularly given that YKL-40 production depends on IL-6 secretion and also IFNγ stimulation, we hypothesized that its expression was higher in patients with cGVHD than in transplanted patients without cGVHD and that it positively correlated with disease severity and activity.

## Patients and methods

### Patients

This case-control study is part of a larger project entitled “Clinical and Biological Factors Determining Severity and Activity of Chronic GVHD After Allogeneic Hematopoietic Stem Cell Transplantation” at the University Hospital Center Zagreb. The project included all patients who were referred to hematologist for post-transplantation follow up, regardless of their age or underlying diagnosis, who consented to the study participation. Excluded from participation were patients with significant medical condition or any other significant circumstance that could affect the patient’s ability to tolerate, comply, or complete the study and patients who according to the investigators assessment had life expectancy less than 3 months. Over the period of July 2013 to October 2015, 76 patients treated with hematopoietic stem cell transplantation (HCST) were included in the project: 47 patients who developed cGVHD and 29 who did not develop cGVHD and who served as controls (Table 1).

**Table 1:** Characteristics of patients who underwent hematopoietic stem cell transplantation (HSCT) with and without chronic graft-vs-host disease (cGVHD)

		cGVHD (n = 35)	Control (n = 21)	*P*
Median age, years (range)		45 (9-60)	40 (16-59)	0.912
Sex	female, n (%)	18 (51.4)	10 (47.6)	0.783
	male, n (%)	17 (48.6)	11 (52.4)	
Diagnosis	aplastic anemia, n (%)	3 (8.6)	1 (4.8)	0.679
	acute lymphoblastic leukemia, acute myeloid leukemia, n (%)	21 (60.0)	10 (47.6)	
	chronic lymphocytic leukemia, n (%)	1 (2.9)	2 (9.5)	
	chronic myeloid leukemia and myeloproliferative diseases, n (%)	6 (17.1)	6 (28.6)	
	lymphoma, n (%)	2 (5.7)	2 (9.5)	
	immunodeficiencies, n (%)	1 (2.9)	0 (0.0)	
	multiple myeloma, n (%)	1 (2.9)	0 (0.0)	
Donor relationship	related, n (%)	21 (60.0)	9 (42.9)	0.213
	unrelated, n (%)	14 (40.0)	12 (57.1)	
Cell source	bone marrow, n (%)	15 (42.9)	8 (38.1)	0.726
	peripheral cells, n (%)	20 (57.1)	13 (61.9)	
Days from transplantation to enrollment, median (range)		463 (61-7853)	428 (190-1770)	0.441

For 56 patients (35 patients with cGVHD and 21 controls) included in the project serum samples were obtained at enrolment and stored. These patients were included in the study presented here. Prior to enrolment all participants signed the informed consent, and the study was approved by the University Hospital Center Zagreb Ethics Committee.

### Data collection

Data regarding the diagnosis, time and type of transplant, and donor characteristics, and demographic data were collected. Blood samples for measurement of YKL-40 level and C-reactive protein (CRP) were taken at the time of study enrollment. For patients with established cGVHD diagnosis additional data regarding the severity and activity of disease were collected using predefined forms. Disease activity was defined by Clinician’s Impression of Activity and Intensity of Immunosuppression Scale. Clinician’s impression of activity was defined as: inactive, off systemic therapy or topical immunosuppression; inactive, on systemic therapy or topical immunosuppression; active irrespective of the level of current therapy; and highly active irrespective of the level of current therapy (47). Intensity of immunosuppression scale was defined as: none; mild = single agent prednisone <0.5 mg/kg/d; moderate = prednisone ≥0.5 mg/kg/d and/or any single agent/modality; high = 2 or more agents/modalities ± prednisone ≥0.5 mg/kg/d (47,48). Disease severity was defined by Global National Institutes of health (NIH) scoring. Patients had mild cGVHD if only 1 or 2 organs (except lungs) were involved, with a maximum score 1 in all affected organs. Patients had moderate cGVHD if at least 1 organ was involved with clinically significant, but not major disability (maximum score 2) or 3 or more organs with no clinically significant functional impairment (maximum score 1 in all affect organs); a lung score 1 was classified as moderate. Patients had severe cGVHD if they had major impairment caused by cGVHD (score 3 in any organ); lung scores of 2 or 3 were classified as severe. Organs scored included the skin, eyes, mouth, gastrointestinal tract, liver, lungs, and joint/fascia. The genital area was scored only in women (49).

### YKL-40 analysis

Plasma samples were prepared from EDTA (EDTA)-anticoagulated blood taken at the time of inclusion and were stored at -80°C until YKL-40 analysis. YKL-40 plasma concentration was measured using a commercially available ELISA kit (R&D Systems Europe, Abingdon, UK).

### Statistical analysis

After testing for normality using Kolmogorov-Smirnov test and due to small sample size we decided to use non-parametric tests. Categorical variables are presented as frequencies and corresponding percentages and quantitative variables as medians and interquartile ranges. The differences in categorical clinical parameters between patients and controls were analyzed using Fisher exact test or Fisher-Freeman-Halton exact test of independence when the contingency table was larger than 2 × 2, while differences in quantitative variables were analyzed using Mann-Whitney U test. Differences in YLK-40 levels between NIH groups were analyzed using Kruskal-Wallis test. Spearman correlation coefficients were calculated to assess the correlation between YLK-40 levels and other clinical variables. *P* values below 0.05 were considered significant. Data analysis software system IBM SPSS Statistics, version 21.0 (IBM Corp., Armonk, NY, USA) was used.

## Results

### Patient characteristics

Median age was 45 years (interquartile range 27-52 years) in the cGVHD group and 40 years (interquartile range 33-54 years) in the control group. There were 18 women in cGVHD group and 9 in the control group. Graft stem cell source was the bone marrow in 15 (42.9%) and 8 (38.1%) and peripheral blood cells in 20 (57.1%) and 13 (61.9%) patients in cGVHD and control group, respectively. 17 (48.6%) and 8 (38.1%) patients received transplant from a related donor, while 18 (51.4%) and 13 (61.9%) patients received it from an unrelated donor in cGVHD and control group, respectively (Table 1). Myeloablative conditioning was used in 17 (48.6%) and 8 (38.1%) patients in cGVHD and control group, respectively. In the cGVHD group, 4 patients had mild, 15 moderate, and 16 severe symptoms as assessed by Global NIH score.

CGVHD and control patient groups were comparable according to age, sex, time from transplantation to enrollment, type of disease, cell source, donor relationship, intensity of conditioning, total body irradiation use in conditioning, and myelofibrosis as primary disease (Table 1).

### Plasma YKL-40 concentration in cGVHD and control group

**Figure 1:**
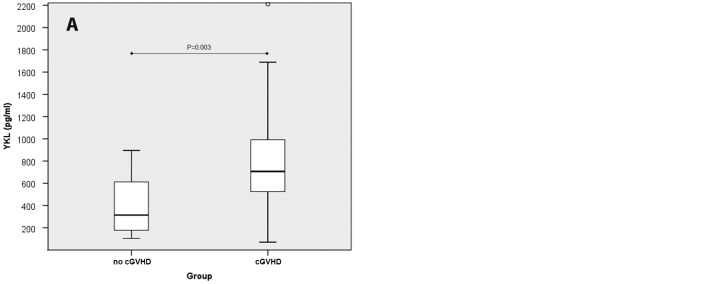
(**A**) Significantly higher YKL-40 serum concentration in chronic graft-vs-host disease (cGVHD) patients compared to control group. (**B**) The difference in YKL-40 serum concentration between cGVHD and control group remained significant after patients suffering from myelofibrosis were excluded from the analysis. 6 patients were excluded, 3 from each group.

YKL-40 levels in cGVHD patients were significantly higher than in the control group (median 707 pg/mL [interquartile range 525-1054] vs 314 pg/mL [interquartile range 169-635]; *P* = 0.003) (Figure 1A). Since YKL-40 is known to be increased in patients suffering from myelofibrosis, we excluded the data of all the patients with a history of myelofibrosis from the analysis (3 patients, [8.6%] in cGVHD group and 3 patients, [14.3%] in control group). After these patients were excluded, the concentration of YKL-40 was still significantly higher in cGVHD group than in the control group (median 633.5 pg/mL [range 71-1689] vs 351.5 pg/mL [range 105-2790]; *P* = 0.017) (Figure 1B).

### Correlation of YKL-40 concentration and disease activity and severity

**Figure 2:**
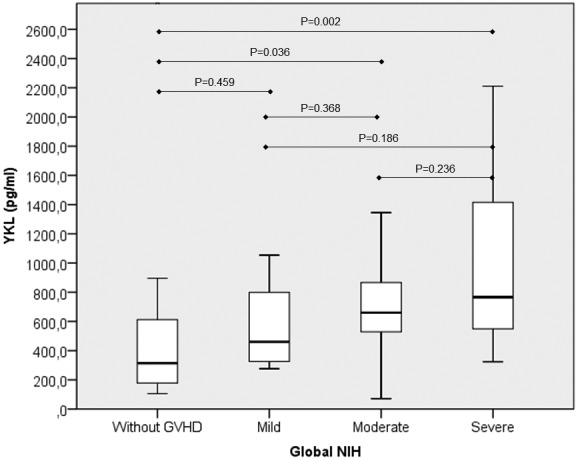
YKL-40 serum concentration was positively correlated with global National Institutes of Health score.

YKL-40 levels significantly positively correlated with Clinician’s Impression of Activity (*P* = 0.016; correlation coefficient 0.412) and Global NIH score (*P* < 0.001; correlation coefficient 0.455) (Figure 2), but not with Intensity of Immunosuppression (*P* = 0.085; correlation coefficient 0.296). Since YKL-40 protein is involved in inflammation process, we tested its correlation with the CRP level, to establish if the YKL-40 level was increased due to persistent chronic inflammation not noticed by the examining physician and found no significant correlation (*P* = 0.581) (Table 2).

**Table 2:** Univariate analysis of YKL-40 serum concentration and chronic graft-vs-host disease (cGVHD) activity and severity measurements

	Correlation coefficient	*P*
Clinicians impression of activity	0.412	0.016
Intensity of immunosuppression	0.296	0.085
Global National Institutes of Health score	0.455	<0.001
National Institutes of Health average score	0.208	0.231
C reactive protein	0.097	0.581

## Discussion

Our study shows that the level of circulating YKL-40 is significantly higher in patients with cGVHD than in transplanted patients without cGVHD and correlates with disease severity and activity, as measured by Global NIH score and Clinicians Impression of Activity, respectively.

Although the exact role of glycoprotein YKL-40 in chronic inflammation is still not elucidated, YKL-40 concentration was found to be increased in 54% of patients with clinically active rheumatoid arthritis (RA). In patients in whom RA became inactive serum YKL-40 concentration decreased after 12 months, but increased in patients with RA flare (50). Furthermore, it has been shown that the level of circulating YKL-40 depends on IL–6 secretion, stimulated by IFNγ (45) and inhibited by IL-4 (33,34,51). Also, IL-6 and IFNγ have been shown to increase during GVHD development (52) and IL-6 is crucial for Th17 pathway (53), while production of IL-4 has been shown to be decreased in cGVHD patients (54). This could in part explain why YKL-40 is increased in active cGVHD reaction.

Furthermore, it was previously shown that patients with myelofibrosis had highly elevated levels of circulating YKL-40 in comparison to healthy controls (43). However, in our study the difference in plasma YKL-40 concentration between cGVHD group and control group remained significant after patients with myelofibrosis were excluded. The cause of elevated YKL-40 in myelofibrosis patients has not been found, but increased bone turnover and pronounced chronic inflammation have been implicated (43). Both processes are aborted after successful HSCT, and therefore, the fact that patient once had myelofibrosis should not influence the post-transplant level of YKL-40 protein. The effect of donor and recipient pre-transplant level of circulating YKL-40 on transplant outcomes (ie, relapse-related mortality, progression-free survival, overall survival, and acute GVHD incidence) has been reported, but post-transplant levels have not been investigated (45,46).

Even though our study distinctly shows concentration of YKL-40 to be significantly higher in patients with cGVHD in comparison to controls, it has several limitations. It is a single center study with a limited number of included patients. Albeit there was no significant difference between groups in time from transplant to sampling, there was a considerably wide range among patients within each group (range for cGVHD group was 61 to 7853 days, control 190 to 1770 days). This is because patients were included at various points after having been diagnosed with cGVHD and at various stages of disease. Even though it would be preferable if the study included only newly diagnosed patients to limit the influence of disease advancement and/or therapy, the patients included in this study were clinically very well characterized according to 2005 NIH Consensus criteria.

In our study YKL-40 levels positively correlated with Global NIH score, a measure of disease severity, and Clinician’s Impression of Activity, a measure of disease activity. We agree that data shown here do not prove YKL-40 protein to be a valid biomarker, but they certainly indicate that it is a strong new candidate. In our opinion, YKL-40 needs to be explored as a potential biomarker on a larger number of cGVHD patients. In addition, a longitudinal study of post-transplant patients, with serial, predefined time points is needed to validate these results, establish the dynamics of YKL-40 expression, and determine its role in evaluating the response to treatment.
